# Pharmacodynamics of Pre-Operative PD1 checkpoint blockade and receptor activator of NFkB ligand (RANKL) inhibition in non-small cell lung cancer (NSCLC): study protocol for a multicentre, open-label, phase 1B/2, translational trial (POPCORN)

**DOI:** 10.1186/s13063-019-3951-x

**Published:** 2019-12-19

**Authors:** Elizabeth Ahern, Annette Cubitt, Emma Ballard, Michele W. L. Teng, William C. Dougall, Mark J. Smyth, David Godbolt, Rishendran Naidoo, Amanda Goldrick, Brett G. M. Hughes

**Affiliations:** 1 Immunology in Cancer and Infection Laboratory, QIMR Berghofer Medical Research Institute, Herston, Queensland Australia; 2 Cancer Immunoregulation and Immunotherapy Laboratory, QIMR Berghofer Medical Research Institute, Herston, Queensland Australia; 3 School of Medicine, University of Queensland, Herston, Queensland Australia; 4 Cancer Care Services, Royal Brisbane and Women’s Hospital, Herston, Queensland Australia; 5 Statistics Unit, QIMR Berghofer Medical Research Institute, Herston, Queensland Australia; 6 Immuno-Oncology Discovery Laboratory, QIMR Berghofer Medical Research Institute, Herston,, Queensland Australia; 7 Department of Pathology, The Prince Charles Hospital, Chermside, Queensland Australia; 8 Department of Surgery, The Prince Charles Hospital, Chermside, Queensland Australia; 9 Department of Haematology and Oncology, Amgen Australia, Kew, Victoria Australia; 10 Cancer Care Services, The Prince Charles Hospital, Chermside, Queensland Australia

**Keywords:** NSCLC, Lung cancer, Immunotherapy, RANKL, Denosumab, PD1, Nivolumab, Neoadjuvant

## Abstract

**Background:**

Neoadjuvant immunotherapy targeting immune checkpoint programmed death-1 (PD-1) is under investigation in various tumour settings including non-small-cell lung cancer (NSCLC). Preclinical models demonstrate the superior power of the immunotherapy provided in a neoadjuvant (pre-operative) compared with an adjuvant (post-operative) setting to eradicate metastatic disease and induce long-lasting antigen-specific immunity. Novel effective immunotherapy combinations are widely sought in the oncology field, targeting non-redundant mechanisms of immune evasion. A promising combination partner with anti-PD1 in NSCLC is denosumab, a monoclonal antibody blocking receptor activator of NF-κB ligand (RANKL). In preclinical cancer models and in a large retrospective case series in NSCLC, anti-cancer activity has been reported for the combination of immune checkpoint inhibition (ICI) and denosumab. Furthermore, clinical trials of ICI and denosumab are underway in advanced melanoma and clear-cell renal cell carcinoma. However, the mechanism of action of combination anti-PD1 and anti-RANKL is poorly defined.

**Methods:**

This open-label multicentre trial will randomise by minimisation 30 patients with resectable stage IA (primary > 2 cm) to IIIA NSCLC to a neoadjuvant treatment regime of either two doses of nivolumab (3 mg/kg every 2 weeks) or two doses of nivolumab (same regimen) plus denosumab (120 mg every 2 weeks, following nivolumab). Each treatment arm is of equal size and will be approximately balanced with respect to histology (squamous vs. non-squamous) and clinical stage (I-II vs. IIIA). All patients will receive surgery for their tumour 2 weeks after the final dose of neoadjuvant therapy. The primary outcome will be translational research to define the tumour-immune correlates of combination therapy compared with monotherapy. Key secondary outcomes will include a comparison of rates of the following between each arm: toxicity, response (pathological and radiological), and microscopically complete resection.

**Discussion:**

The POPCORN study provides a unique platform for translational research to determine the mechanism of action of a novel proposed combination immunotherapy for cancer.

**Trial registration:**

Prospectively registered on Australian New Zealand Clinical Trials Registry (ACTRN12618001121257) on 06/07/2018.

## Background

In early stage and selected locally advanced non-small cell lung cancer (NSCLC), surgical excision is recommended where possible (stage I, II and selected IIIA). Surgery improves cure in stage 1–2 disease [1]. Unfortunately, evidence suggests that fewer than 30% of NSCLC patients receive surgery because of factors such as tumour stage (i.e., locally-advanced unresectable or metastatic disease at presentation) or physiologic unsuitability for the required surgery [2–4]. Furthermore, survival after surgical excision of early-stage NSCLC remains suboptimal and is stage-dependent [5]. Adjuvant chemotherapy is offered to selected patients, and when indicated, it improves the absolute survival rates by approximately 5% [6, 7]. In chemotherapy trials, neoadjuvant approaches have broadly equivalent efficacy but a higher rate of completion of all planned cycles [8, 9]. Major pathological response (MPR), indicating ≤10% viable tumour remaining at the time of surgery, is significantly correlated with improved survival after the administration of neoadjuvant chemotherapy in NSCLC [10].

More recently, immunotherapy blocking immune checkpoints expressed by T-lymphocytes (immune checkpoint inhibition, ICI) demonstrated superior outcomes compared with chemotherapy in various settings in advanced NSCLC [11–15]. Such treatment aims to reverse tumour-mediated immune suppression, which leads to dysfunctional tumour-associated immune cells. Furthermore, in mouse cancer models, neoadjuvant immunotherapy demonstrated superior power to eradicate micro-metastatic disease and improve survival when compared with adjuvant immunotherapy [16]. The mechanism involves induction of long-lasting antigen-specific T-cell memory [16]. In 2018, a small phase II trial showed that two doses of neoadjuvant nivolumab (antibody blocking programmed death-1, PD1) in patients with resectable NSCLC resulted in an impressive rate of pathological response (45% major pathological response) and was well-tolerated [17]. This rate of major pathological response is higher than that reported historically for neoadjuvant chemotherapy in similar patient groups (22%) [10], albeit with a small patient cohort. In that trial, effective neoadjuvant immunotherapy was associated with higher clonality of tumour-infiltrating T-lymphocytes and an induction and expansion of tumour antigen-specific T-lymphocytes detected in peripheral blood [17]. Pathologically, tumour regression was accompanied by marked signs of local immune activity, including the influx of lymphocytes and macrophages and the creation of tertiary lymphoid structures along with tumour-cell death [18]. Subsequently, preliminary outcomes of further early phase trials of neoadjuvant nivolumab or atezolizumab (antibody blocking PD1-Ligand 1, PD-L1) with or without chemotherapy have been reported, with these therapies proving well-tolerated and showing considerable evidence of clinical efficacy [19–21].

Receptor activator of NF-kB ligand (RANKL) (TNFSF11A) is a member of the tumour necrosis factor superfamily. Denosumab (Amgen, Inc.) is a fully-human IgG2 monoclonal antibody blocking RANKL and is approved by the United States Food and Drug Administration for various malignant and non-malignant indications, including bone metastases and osteoporosis, given its anti-osteoclastic activity [22]. The receptor for RANKL, RANK (TNFSFR11), is expressed by osteoclasts and other cells of the monocyte-macrophage lineage, while RANKL is expressed by activated T cells [23]. In several preclinical cancer models, the blockade of RANKL improved the efficacy of various ICI, including anti-PD1; of note, RANKL blockade alone had minimal single agent efficacy [24–26]. Some exceptional anti-tumour responses have been reported in case reports following concurrent ICI and denosumab administration in patients with advanced melanoma, and small clinical retrospective series have reported encouraging response rates with similar denosumab-ICI combinations in advanced melanoma [27]. Two phase II trials are underway to assess the efficacy of denosumab with various ICI in advanced melanoma (NCT03280667) and advanced clear cell renal cell carcinoma (NCT03161756). However, these trials do not include an ICI-alone comparator arm, which will complicate interpretation with respect to the magnitude and mechanism of the effect of blocking RANKL in addition to ICI in cancer.

The totality of evidence suggests that NSCLC is a good tumour type in which to explore this unanswered mechanistic question. RANK can be expressed by human lung cancer cells, and higher RANKL gene expression is significantly correlated with poorer survival in published human NSCLC datasets drawing from large mRNA expression datasets [28]. Some clinical evidence suggests that blocking RANKL with denosumab may improve survival in patients with advanced NSCLC, independently of its effects on bone metastases. Post-hoc survival analysis of a large subset of patients with NSCLC (*n* = 702) in a larger randomised phase III trial which compared denosumab with zoledronic acid (an alternative anti-resorptive agent active in skeletal metastases) revealed a significant benefit of denosumab (HR for survival 0.80, 95% CI 0.67–0.95, *p* = 0.01) [29, 30]. In a relatively large retrospective case series of patients with advanced NSCLC, the duration of concurrent denosumab therapy with ICI was associated with significantly improved survival in advanced NSCLC [27].

Taken together, evidence suggests that neoadjuvant immunotherapy comprises a promising approach in NSCLC, and that anti-RANKL may prove a rational combination partner with ICI in this indication. Furthermore, the conduct of neoadjuvant trials is particularly favourable for translational research, as baseline biopsies followed by systemic therapy and then surgery allow easy access to pre- and post-treatment tissue and blood samples for correlative testing. The mechanism of action, efficacy and safety of denosumab and nivolumab (compared with nivolumab alone) in the neoadjuvant treatment of NSCLC will be assessed in POPCORN: Preoperative PD1 checkpoint blockade and receptor activator of NFkB ligand (RANKL) inhibition in non-small cell lung cancer (NSCLC) (ACTRN12618001121257). This study was funded by an education grant provided by Amgen. Conduct of the study and all analysis will be performed by the investigators independently of Amgen.

## Methods/Design

### Study aim

POPCORN is a signal-seeking study to provide information about the magnitude of pharmacodynamic effect, activity (clinical/pathological response) and safety of combination anti-RANKL (denosumab) and anti-PD1 (nivolumab) compared with anti-PD1 (nivolumab) alone in the preoperative treatment of resectable NSCLC.

### Study population

A total of 30 patients with NSCLC will be enrolled in this study. Eligible patients will be adults with stage IA (> 2 cm)-IIIA non-small cell lung cancer who have been deemed suitable for up-front curative treatment via surgery by a cardiothoracic surgeon as part of a lung cancer multidisciplinary team. Potential study participants will be identified through the lung cancer multidisciplinary teams at participating trial sites.

### Inclusion and exclusion criteria

Inclusion criteria were as follows: adult patients with histological or cytological diagnosis of NSCLC with 8th edition UICC/AJCC 2017 stage I, II or IIIA NSCLC (tumour ≥2 cm diameter) as assessed by staging investigations including by FDG-PET scan (with strong encouragement to pathologically confirm the status of suspected N2 nodes); measurable primary tumour on CT scan (3-mm slice thickness) per RECIST criteria; sufficient baseline histological specimen from primary tumour (and locoregional lymph node metastasis if clinically suspected) available for translational research; no prior therapy for NSCLC; ECOG performance status 0–1; and adequate organ function. To be eligible for the study, patients must be considered to have potentially resectable disease on the basis of the preoperative investigations. They must be physiologically suitable for surgery, and the expected post-surgical FEV1 must be at least 1 L.

Exclusion criteria were as follows: patients with small cell or mixed small cell histology subtypes; prior malignancy within 5 years (other than non-melanoma skin cancer or adequately treated stage I in situ cervical cancer); recent receipt of another investigational drug or anti-cancer treatment; any prior treatment with therapies targeting T-cell immune checkpoint pathways or denosumab; active, known or suspected autoimmune disease (with certain exceptions such as vitiligo or type 1 diabetes mellitus); use of corticosteroids (at a dosage equivalent of 10 mg prednisolone per day or higher); contraindication to corticosteroids; and concomitant medical or psychiatric disorders which would compromise the patient’s safety or participation (including dental disorders such as pre-existing osteonecrosis of the jaw, recent live vaccine, or serious hypersensitivity to trial drugs).

### Study design

POPCORN is an open-label, multi-centre phase 1B/2 study with a pharmacodynamic endpoint. It will be conducted in four centres in Australia. Randomization by minimisation is performed at the Statistics Unit of Queensland Institute of Medical Research with stratification by histology (squamous vs. non-squamous) and tumour stage.

**Fig. 1:**
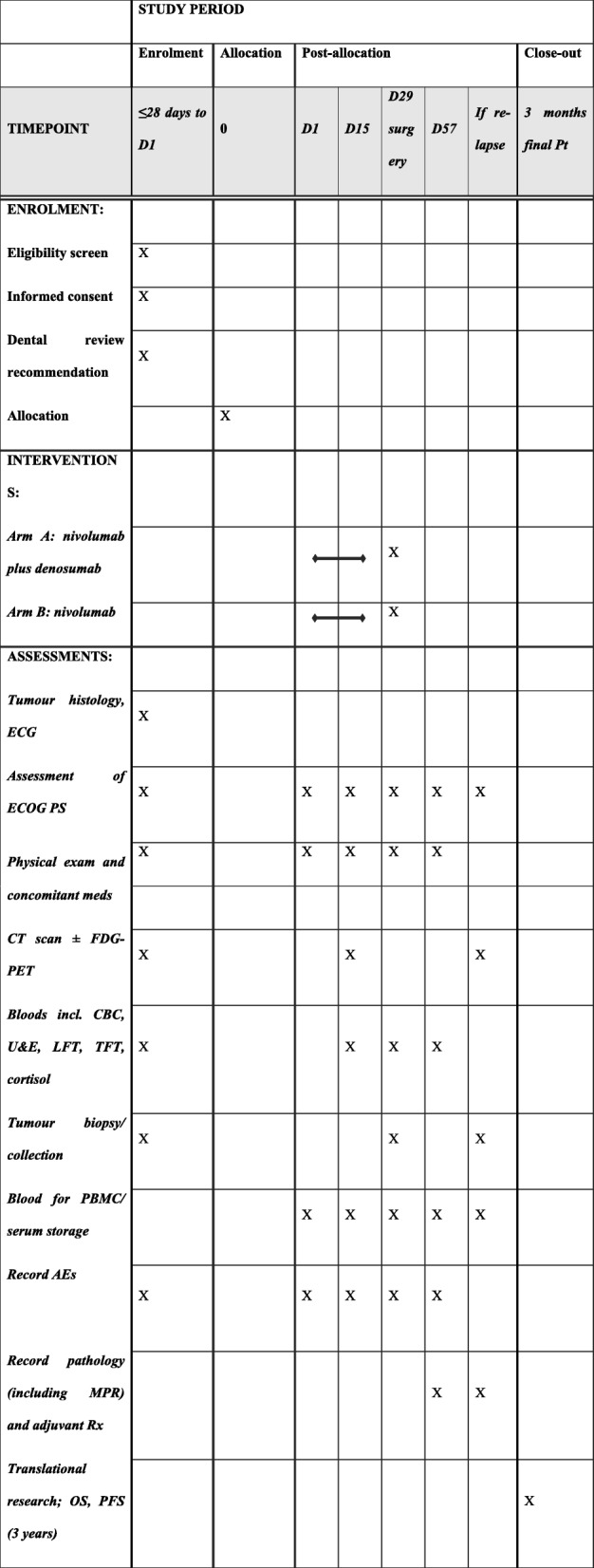
SPIRIT schedule of enrolment, interventions and assessments. *ECG* electrocardiogram, *ECOG* PS Eastern Cooperative Oncology Group Performance Status, *CT* computed tomography, *FDG-PET* fluorodeoxyglucose-position emission tomography, *CBC* complete blood count, *U&E* urea and electrolytes, *LFT* liver function test, *TFT* thyroid function test, *PBMC* peripheral blood mononuclear cells, *AE* adverse events, *MPR* major pathological response, *Rx* treatment, *OS* overall survival, *PFS* progression-free survival

**Fig. 2:**
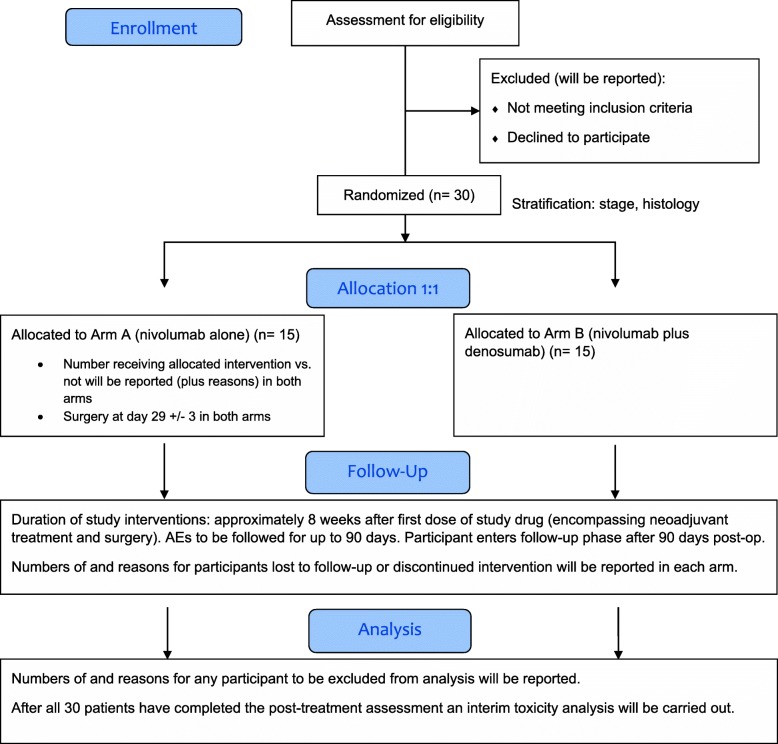
CONSORT diagram of the POPCORN study

The schedule of study assessments is shown in Fig. 1. The CONSORT diagram for the trial is shown in Fig. 2 [31]. The SPIRIT checklist is shown in Additional file 1.

A subject will have completed the study interventions approximately 8 weeks after the first dose of study drug (encompassing neoadjuvant treatment and surgery). All AEs will be followed up for a maximum of 90 days after the final dose of study drug; therefore, the subject is considered as entering the survival follow-up phase after 90 days post-surgery. Subsequently, patients will be followed according to the institution’s standard practice. The close-out date of the trial will be 3 months after surgery for the final randomized participant, but with a further 3-year follow-up after the end of accrual to record long-term survival outcomes. Any adjuvant treatment, date and site of progression, date of death and cause of death will be recorded.

Ongoing clinical review of study participants in the follow-up phase will be at 3-month intervals for 3 years, with restaging scans (CT and/or FDG-PET) per institutional practice. Outcome assessments will continue for a total of 3 years post-surgery.

### Interventions

**Fig. 3:**
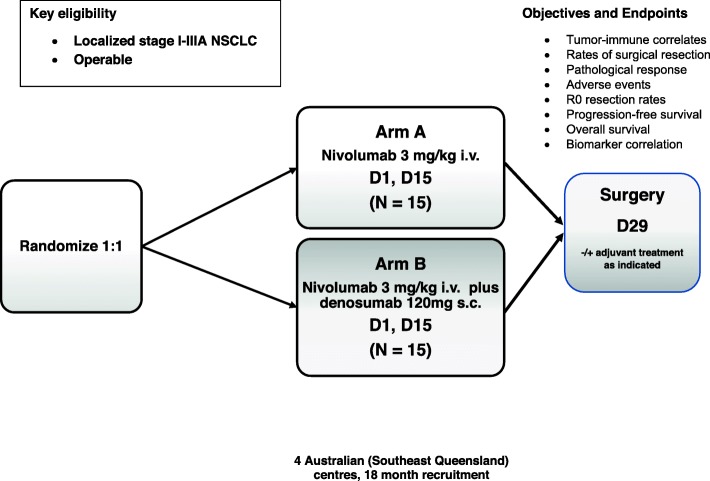
POPCORN study schema. *NSCLC* non-small cell lung cancer, *i.v.* intravenous, *s.c.* subcutaneous

Neoadjuvant systemic therapy will occur on two separate occasions, 2 weeks apart. In arm A, on each occasion participants will receive nivolumab (3 mg/kg i.v.), whereas in arm B, participants will receive nivolumab (3 mg/kg i.v.) and denosumab (120 mg s.c.) (Fig. 3). All patients in arm B will also receive calcium and vitamin D supplementation unless hypercalcemia is present, and hypocalcemia must be corrected prior to initiating therapy.

Surgery should be carried out on day 29 (± 3 days) of the study (2 weeks after the second dose of nivolumab +/− denosumab). The surgical operation to remove the primary tumour should be lobectomy, pneumonectomy or anatomical segmentectomy and other surgery as required. Thoracoscopic surgical techniques are permitted. Wedge resection or non-anatomical surgical dissection is not permitted. Surgery should also include appropriate mediastinal lymph node sampling or dissection and macroscopic margins of ≥2 cm and microscopic margins of ≥1 cm being the aim.

All patients should be offered appropriate adjuvant therapy as per institutional practice according to the recommendations of treating clinicians, preferably based on a multidisciplinary team review. This therapy is strongly recommended to consist of four cycles of a platinum doublet chemotherapy (common regimen consisting of cisplatin 50 mg/m^2^ days 1 and 8 and vinorelbine 25 mg/m^2^ days 1, 8, 15 +/− 22 every 4 weeks for four cycles). Adjuvant chemotherapy should be considered in patients with pre-study nodal involvement (N1 or N2), a primary tumour > 4 cm and at the discretion of the treating investigator. Post-operative radiotherapy should also be considered in patients with pathologically confirmed N2 nodal involvement or positive surgical resection margins (R1 disease).

### Intervention safety monitoring and assessment

Adverse events (AE), defined as any untoward medical occurrence(s) in a trial participant regardless of causality with trial interventions, will be systematically monitored and recorded. These will be classified and graded according to the National Cancer Institute Common Terminology Criteria for Adverse Events version 4.03 (NCI CTCAE v4.03). Serious AEs (SAE) will be reported to the appropriate ethics committees and competent authorities as well as the study safety committee. A suspected unexpected serious adverse reaction (SUSAR), which is an unexpected SAE related to the intervention, will additionally be reported to the drug manufacturer. If the AE is deemed by the investigator to have been caused, or probably caused, by the investigational treatment (nivolumab and/or denosumab), this will be labelled a TRAE (treatment-related adverse event).

Study participants will be reviewed clinically for the presence of AEs by the site investigator prior to each cycle of neoadjuvant therapy, prior to surgery, and 4 weeks following surgery. Blood tests, including complete blood count, liver and renal function tests, electrolytes, thyroid function tests and serum cortisol will be reviewed at these visits. Immune-related adverse events are managed following algorithms as compiled by expert groups or as available in the product information [32]. Re-challenge after a suspected TRAE is permitted provided symptoms resolve to an appropriate level to meet criteria to resume treatment and do not meet any of the permanent discontinuation criteria (such as grade 4 and select grade 3 toxicities). Delay in dose 2 administration of up to 5 days is permitted, but further delays are not permitted, as this could unreasonably delay surgery. Dose reductions and dose escalations are not permitted. An independent data safety monitoring committee will monitor the conduct and safety of the trial during recruitment.

A pregnancy test is mandated for women of childbearing age within 72 h of commencing treatment and must be negative. Patients on study with reproductive potential, or female partners with reproductive potential, must use an effective contraceptive method during the trial and for 3 months after the completion of chemotherapy.

All patients are recommended to have a dental examination prior to commencing denosumab, maintain good oral hygiene while on denosumab, and avoid invasive dental procedures during treatment with denosumab and for at least a month after the final dose of denosumab. If osteonecrosis of the jaw is suspected, treatment with denosumab is halted, and the patient assessed by a dentist or oral surgeon.

### Endpoints

The primary endpoint is to define pharmacodynamic correlates of neoadjuvant therapy for each arm (combination anti-RANKL and anti-PD1 compared with anti-PD1 alone) in NSCLC. This is a signal-seeking study for this endpoint. Pharmacodynamic correlates will include the following parameters assessed in the tumour and, where relevant, the blood:

- T-cell receptor (TCR) clonality, comparing baseline and on−/post-treatment samples
- RNA/transcription profile changes for immune cells (such as infiltrating T cells and myeloid cells) and other cells of interest (such as tumour cells), to define treatment response
- Analysis of genomic alterations, including estimation of expressed mutation-associated neoantigen load, and association with treatment response, using Nanostring Immune Profiling Panel (from FFPE and/or fresh tissue-derived RNA) and whole-genome sequencing techniques
- Expression of markers of interest via multiplex immunohistochemistry (formalin-fixed, paraffin-embedded tumour samples) and/or flow cytometry (fresh, dissociated tumour tissue or peripheral blood mononuclear cells) such as the expression of target proteins RANKL and immune cell phenotypic markers

**Fig. 4:**
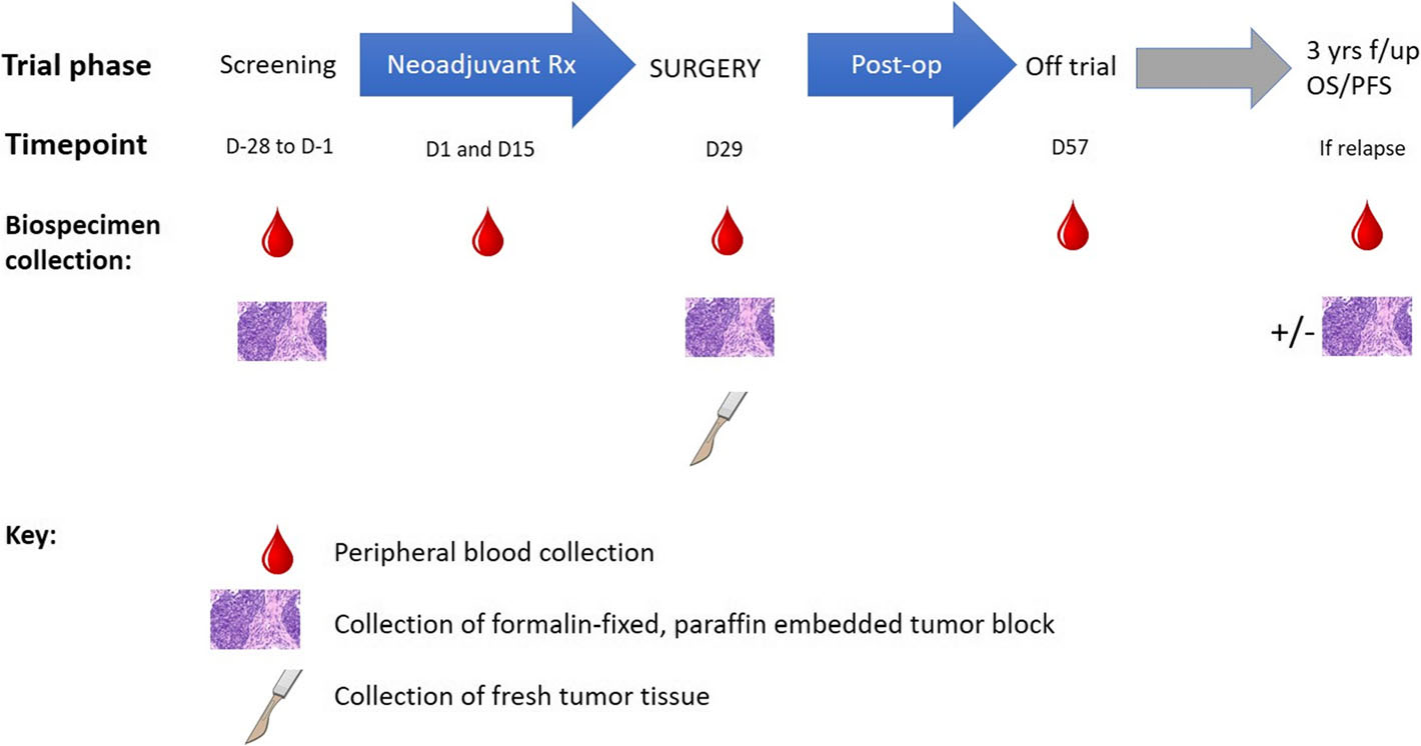
Translational research schedule for collection of POPCORN trial biospecimens. Figure acknowledgements: FFPE by Librepath - Own work, CC BY-SA 3.0, https://commons.wikimedia.org/w/index.php?curid=45097146; scalpel by Petit B - Own work, CC BY-SA 4.0, https://commons.wikimedia.org/w/index.php?curid=41650124

The schedule for biobanking of clinical materials is shown in Fig. 4. Formalin-fixed, paraffin-embedded tumour tissue will be stored at baseline (pre-treatment) and surgical timepoints, with participants strongly encouraged to consent for tumour biopsy for translational research purposes in the event of recurrent or metastatic disease. The collection of non-fixed tumour, including nodal metastases, will be performed where feasible with the assistance of the trial site surgeon and pathologist. The fresh materials will be transported to the central laboratory (within 2 hours of the operation where possible) and dissociated and processed on the same day. Blood will be collected at baseline, before each neoadjuvant systemic treatment, at the time of surgery, at the post-surgical visit, and in the event of tumour recurrence. This blood will be processed in the central trial laboratory, for the storage of serum and peripheral blood mononuclear cells.

Secondary and exploratory endpoints are defined as follows, and will be compared between arms:

- The proportion of patients with evidence of pathological response as determined by blinded central pathology review, including major pathological response (MPR) (defined as <10% viable tumour cells in the resected specimen)
- Response by CT scanning, with the timepoint response assessed per RECIST v1.1 for the comparison of baseline and pre-operative scans
- Safety, according to the following:
  - ◦ Rates of NCI CTCAE v4.03 grade 3–4 toxicity will be assessed by comparing the proportion of patients in each arm who are noted to have an AE of grade 3–4, regardless of causality.
  - ◦ Proportion of patients in each arm who receive planned surgery without delay due to TRAE will be compared. If the surgery cannot proceed due to an unforeseen factor that was deemed by the investigator to probably not be caused by the investigational treatment, this will be recorded but will not be deemed delay due to TRAE. A delay is defined as an event where the surgery is rescheduled for > 24 h later than originally planned due to clinical considerations.
- Feasibility of approach, as reflected in the R0 resection rate, will be assessed based upon the operative and pathological report arising from the surgical resection. An R0 resection indicates that there is a microscopically and macroscopically negative margin.
- As an exploratory endpoint, progression-free survival (PFS) (defined as time between randomization and first evidence of disease progression or death from any cause) and overall survival (OS) (defined as time between randomization and death from any cause) will be recorded for trial participants in each group, for 3 years of follow-up or the latest available data, whichever is longer.

### Statistical analysis

As a signal-seeking study, the emphasis of this project is on gaining an understanding of the mechanism of action, activity and safety of combination neoadjuvant therapy with denosumab and nivolumab. The sample size was based on estimated patient numbers available during the study period (18 months). The comparison of interest is between the combination neoadjuvant therapy compared to nivolumab alone. Categorical variables will be summarised as the frequency and percentage, and continuous variables, as the mean and standard deviation or median and interquartile range. A 95% confidence interval will be reported for primary outcomes. Categorical variables will be examined using the Pearson chi-squared test or Fisher’s exact test when more than 20% of the expected value are less than 5. Continuous variables will be examined using the Student t-test or Mann-Whitney U test if the data are not normally distributed. Paired data will be examined using a paired t-test or repeated measures analysis of variance (ANOVA). Kaplan-Meier will be used to estimate progression-free survival and overall survival, with the log-rank test being used to assess differences between treatments. *P* values less than 0.01 will be considered significant.

The intention-to-treat population will be all patients with NSCLC who are randomized to the study. The as-treated population will be evaluable for toxicity and will include all patients who receive at least one dose of study therapy. After all 30 patients have completed the post-treatment assessment, an interim toxicity analysis will be carried out. An interim analysis may also be undertaken for exploratory laboratory investigations on normal and tumour tissue.

### Dissemination of results

The study results based on the trial data will be released to the participating physicians, referring physicians, patients and the general medical community. During study close-out, an interim period will be used to complete data collection, following which the manuscript(s) based on the trial results will be submitted to peer-reviewed journals. Authorship criteria as defined by the International Committee of Medical Journal Editors will be followed.

## Discussion

Neoadjuvant trials provide a unique and valuable opportunity in translational research. In addition to providing pharmacodynamic information about the mechanism of action of the neoadjuvant approach, these trials can explore other important aspects such as questions about biomarkers for response or resistance and the expression of novel pathway markers and their modification over the time course of treatment. With respect to POPCORN, neoadjuvant immunotherapy in cancer has a sound preclinical rationale, and other early-phase trials of neoadjuvant ICI with similar design are demonstrating impressive results in NSCLC. Given the well-characterised toxicity profile of denosumab, the investigators anticipate minimal additive AE with nivolumab; in particular, immune-related AEs are not associated with denosumab [22, 23]. POPCORN is a small trial which is intended to assess the mechanism of action of a novel immunotherapy combination and will allow an estimation of the magnitude of any incremental improvement in efficacy. If such improvement is observed, this could help in the design of larger trials in the future.

### Trial status

The first trial protocol and the patient information and consent form were submitted to the Human Research Ethics Committee on 30 July 2018, and amended documents (version 2.0) were approved on 6 September 2018. These were last updated on 4 December 2018. The trial is open as of May 2019, and the first patient was enrolled and began treatment in August 2019. Recruitment is ongoing.

## Supplementary information

**Additional file 1.** SPIRIT 2013 Checklist: Recommended items to address in a clinical trial protocol and related documents*.

## Data Availability

Not applicable

## Abbreviations

AE: adverse event
ECOG: Eastern Cooperative Oncology Group
ICI: immune checkpoint inhibitor
NCI CTCAE: National Cancer Institute Common Terminology Criteria for Adverse Events
NSCLC: non-small cell lung cancer
PD1: programmed death-1
RANKL: receptor activator of NF-κB
RNA: ribonucleic acid
SAE: serious adverse event
SUSAR: suspected unexpected serious adverse reaction
TCR: T-cell receptor
TRAE: treatment-related adverse event
UICC/AJCC: Union for International Cancer Control/American Joint Committee on Cancer
